# Detection of pH and Enzyme-Free H_2_O_2_ Sensing Mechanism by Using GdO_*x*_ Membrane in Electrolyte-Insulator-Semiconductor Structure

**DOI:** 10.1186/s11671-016-1657-5

**Published:** 2016-09-29

**Authors:** Pankaj Kumar, Siddheswar Maikap, Jian-Tai Qiu, Surajit Jana, Anisha Roy, Kanishk Singh, Hsin-Ming Cheng, Mu-Tung Chang, Rajat Mahapatra, Hsien-Chin Chiu, Jer-Ren Yang

**Affiliations:** 1 Department of Electronic Engineering, Chang Gung University (CGU), 259 Wen-Hwa 1st Rd., Kwei-Shan, Tao-Yuan, 333 Taiwan; 2 Bio-Sensor Lab., Biomedical Engineering Research Center, Department of Electronic Engineering, Chang Gung University, Tao-Yuan, 333 Taiwan; 3 Center for Reliability Science and Technologies (CReST), Department of Electronic Engineering, Chang Gung University, Tao-Yuan, 333 Taiwan; 4 Department of Biomedical Sciences, School of Medicine, Chang Gung University (CGU), Tao-Yuan, 333 Taiwan; 5 Division of Gyn-Oncology, Department of Obs/Gyn, Chang Gung Memorial Hospital (CGMH), Tao-Yuan, 333 Taiwan; 6 Material and Chemical Research Laboratories (MRL), Industrial Technology Research Institute (ITRI), Hsinchu, 195 Taiwan; 7 Department of Electronics and Communications Engineering, National Institute of Technology (NIT), Durgapur, 713209 India; 8 Department of Materials Science and Engineering, National Taiwan University (NTU), Taipei, 106 Taiwan

**Keywords:** Enzyme-free H_2_O_2_, pH detection, GdO_*x*_, Sensing mechanism, Catalytic, EIS structure

## Abstract

A 15-nm-thick GdO_*x*_ membrane in an electrolyte-insulator-semiconductor (EIS) structure shows a higher pH sensitivity of 54.2 mV/pH and enzyme-free hydrogen peroxide (H_2_O_2_) detection than those of the bare SiO_2_ and 3-nm-thick GdO_*x*_ membranes for the first time. Polycrystalline grain and higher Gd content of the thicker GdO_*x*_ films are confirmed by transmission electron microscopy (TEM) and X-ray photo-electron spectroscopy (XPS), respectively. In a thicker GdO_*x*_ membrane, polycrystalline grain has lower energy gap and Gd^2+^ oxidation states lead to change Gd^3+^ states in the presence of H_2_O_2_, which are confirmed by electron energy loss spectroscopy (EELS). The oxidation/reduction (redox) properties of thicker GdO_*x*_ membrane with higher Gd content are responsible for detecting H_2_O_2_ whereas both bare SiO_2_ and thinner GdO_*x*_ membranes do not show sensing. A low detection limit of 1 μM is obtained due to strong catalytic activity of Gd. The reference voltage shift increases with increase of the H_2_O_2_ concentration from 1 to 200 μM owing to more generation of Gd^3+^ ions, and the H_2_O_2_ sensing mechanism has been explained as well.

## Background

Recently, hydrogen peroxide (H_2_O_2_) is a major intermediate of biological cycles which has been used as a potential biomarker for oxidative stress diagnosis as well as a major catalyst for immune sensing [1, 2]. On the other hand, it is also an essential compound of bleach industries and waste water treatment. H_2_O_2_ has a major role in modulating mitochondrial function by inhibiting activities of the mitochondrial enzyme in a fully reversible fashion [3, 4]. The H_2_O_2_ sensing assay relies on the use of the enzyme horse radish peroxidase (HRP) to oxidize its substrates and detection using spectrophotometer [5]. H_2_O_2_ sensing in a simple way, with a short time detection with high specificity, is demanded for future disease diagnosis of the human body, and enzyme-free electro-catalytic methods have gained the attention for H_2_O_2_ sensing. Therefore, various catalysts such as metal, metal oxides, and redox polymers have been reported to detect H_2_O_2_ [6–12]. Huang et al. [13] have used the glassy carbon electrode modified by Si nanowire-dispersed CuO nanoparticle. Maji et al. [14] have demonstrated an amperometric H_2_O_2_ sensor based on reduced graphene oxide-coated silica modified with Au nanoparticles. Wang et al. [15] have developed a H_2_O_2_ sensor by using MoS_2_ nanoparticles. Sun et al. [16] have reported a dumbbell-like Pt-Pd-Fe_3_O_4_ nanoparticle-modified glassy carbon electrode which shows electro-catalytic reduction. Liu et al. [17] have reported an amperometric H_2_O_2_ sensor based on a Si substrate modified with carbon nanotube microelectrode coated by Pd nanoparticles. Kong et al. [18] have reported a non-enzymatic H_2_O_2_ sensor based on a Co_3_O_4_ nanowire grown over a reduced graphene oxide sheet. Hao et al. [19] have developed an amperometric H_2_O_2_ sensor based on Fe_2_O_3_ nanoparticles. Bai et al. [20] have reported a sensor based on carbon dot-decorated multi-walled carbon nano-composites. Silver (Ag) nanowire [21] and nanoparticle-decorated graphene [22] have been also reported for H_2_O_2_ sensing. Most of the above groups have used different materials using cyclic voltammetry/amperometric methods to sense H_2_O_2_ (ranging from few nanomolars to millimolars) due to different oxidation states in the presence of H_2_O_2_. On the other hand, high-k materials such as Al_2_O_3_ [23], Ta_2_O_5_ [24], and HfO_2_ [25] in an electrolyte-insulator-semiconductor (EIS) structure have been reported for pH sensing only; however, the Gd_2_O_3_ materials that have been reported are few [26, 27], and even then, there is no report for enzyme-free H_2_O_2_ sensing by using a GdO_*x*_ (*x* < 1.5) material in a simple EIS structure. In this paper, detection of a pH and enzyme-free H_2_O_2_ sensing mechanism has been investigated by using a GdO_*x*_ membrane in a simple EIS structure for the first time. Polycrystalline grain, Gd content, and oxidation states (Gd^2+^/Gd^3+^) have been confirmed by transmission electron microscope (TEM), X-ray photo-electron spectroscopy (XPS), and electron energy loss spectroscopy (EELS) on grain and boundary regions. The 15-nm-thick GdO_*x*_ membrane detects H_2_O_2_ whereas both 3-nm-thick GdO_*x*_ and bare SiO_2_ membranes do not sense H_2_O_2_. Due to the strong catalytic activity of Gd, a low detection limit of 1 μM is obtained. Both time- and concentration-dependent H_2_O_2_ sensing and its mechanism have been investigated.

## Methods

**Fig. 1:**
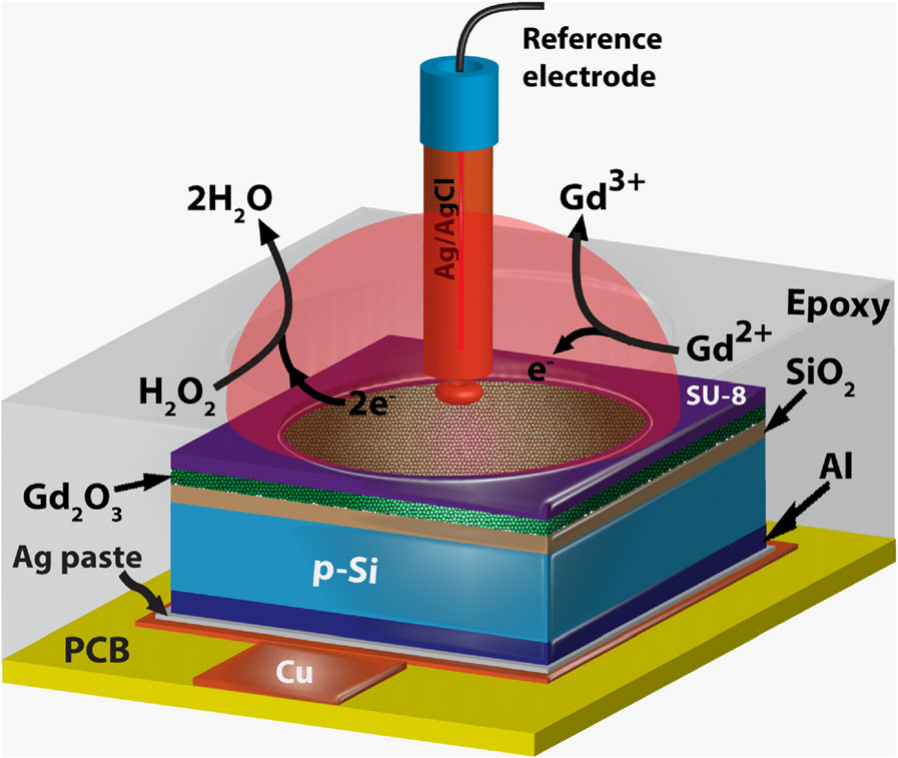
Schematic view of our pH and H_2_O_2_ sensor using Gd_2_O_3_ (or GdO_*x*_ (*x* < 1.5)) membrane and demonstration of H_2_O_2_ sensing mechanism

p-type 4-in. Si (100) wafer was cleaned by the Radio Corporation of America (RCA) process. Prior to thermal growth of SiO_2_, HF dip was used to remove native oxide from the surface. After the cleaning process, a 40-nm-thick SiO_2_ layer was grown as an insulating layer by dry oxidation process at 950 °C. Then, the back-side-grown SiO_2_ layer was removed by using a buffer oxide etching (BOE) solution. To fabricate the EIS chip, a 300-nm-thick Al film was deposited on the back side of the Si wafer. The sensing membrane area was defined by standard photolithography process using a negative photoresist-SU8. Then, EIS devices were attached on a printed circuit board having copper lines. An epoxy layer was used to encapsulate the EIS structure and the copper line. Therefore, a sensor (S1) using SiO_2_ membrane was fabricated. Our fabrication process of EIS structure can be found elsewhere [28]. This SiO_2_ sensing membrane was modified by deposition of 3-nm- (S2) and 15-nm-thick (S3) GdO_*x*_ films. The GdO_*x*_ film was deposited by electron beam evaporation. The Gd_2_O_3_ granules were used during deposition, and the deposition rate was 6 nm/min. A schematic view of the Gd_2_O_3_- (or GdO_*x*_ (*x* < 1.5)) modified SiO_2_ sensor is shown in Fig. 1. To probe the thickness and microstructure of GdO_*x*_ films, low-voltage spherical aberration corrected field emission TEM (Cs-corrected FE-TEM) was performed. The model number is JEOL JEM-ARM200F with accelerating voltages of 60, 120, and 200 kV. In addition, a Cs-corrected FE-TEM Oxford energy spectrometer (energy-dispersive spectroscopy, EDS) and electron loss EDS (EELS, Model 965 QuantumER^TM^) were used to observe the elemental composition on polycrystalline grain and boundaries. The ambient temperature of our laboratory was 21 ± 3 °C and relative humidity was 50 ± 10 %. The elemental composition was investigated by XPS analyzing chamber. The vacuum of the XPS chamber was 1 × 10^−9^ Torr. The spectra were recorded by using an Al K∝ monochrome X-ray at an energy of 1486.6 eV. The scanning energy range from 0 to 1350 eV was used. All spectra were calibrated by C*1s* spectrum at a centered peak energy of 284.6 eV. After depositing the GdO_*x*_ films on the SiO_2_/Si substrates, the samples were transferred immediately to the XPS chamber. The capacitance-voltage (C-V) measurements were performed by using Agilent 4284A LCR meter and an Ag/AgCl reference electrode was used. The measurement frequency was 100 Hz. The sweep voltage was applied on the Ag/AgCl electrode. The reference voltage (V_r_) was measured at 50 % of accumulation capacitance.

## Results and Discussion

**Fig. 2:**
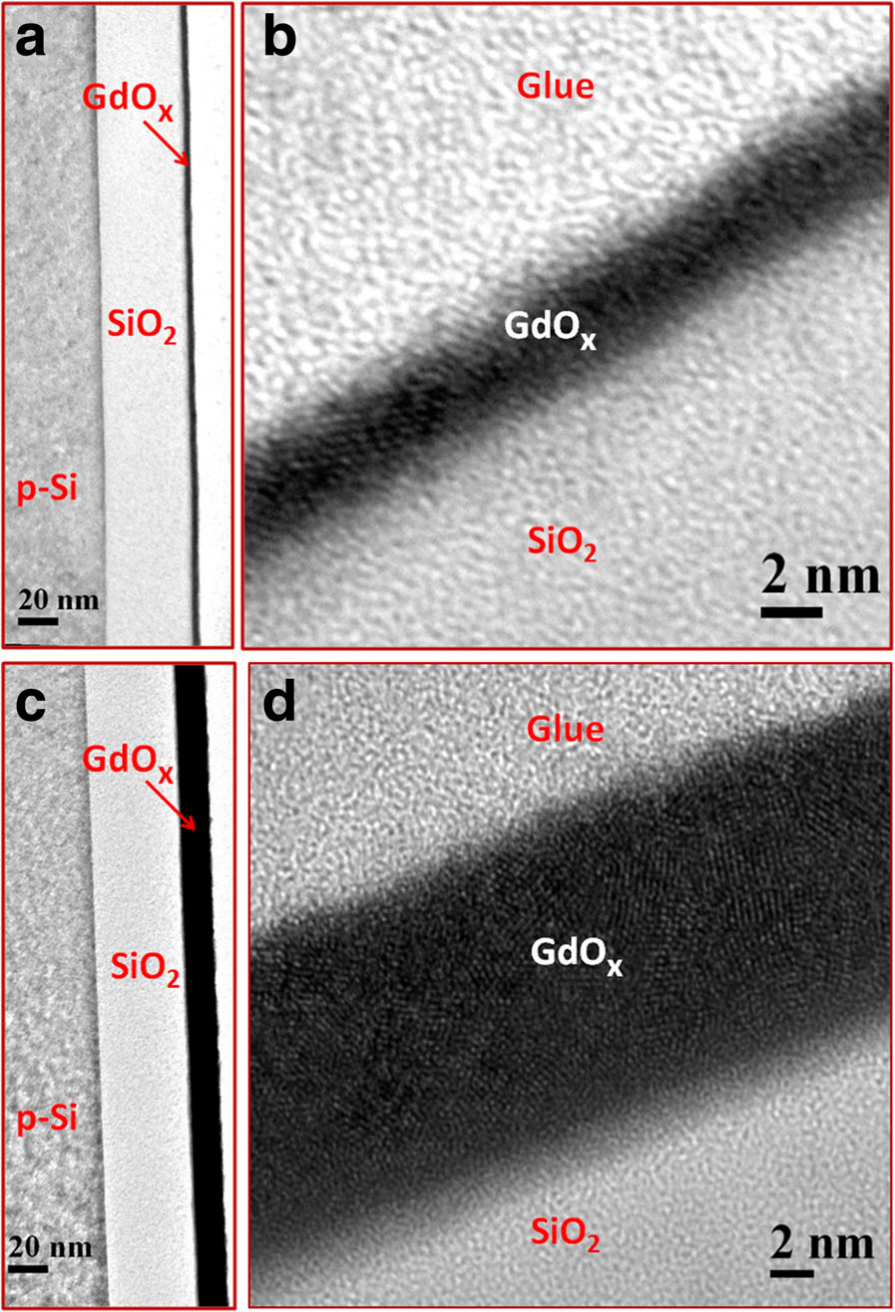
TEM images of **a** 3-nm-thick GdO_*x*_ membrane on 40-nm-thick SiO_2_ layer (S2) and **b** zoom in view of a. TEM images of **c** 15-nm-thick GdO_*x*_ membrane (S3) and **d** zoom in view of c. Thicker membrane shows clear polycrystalline grain

**Fig. 3:**
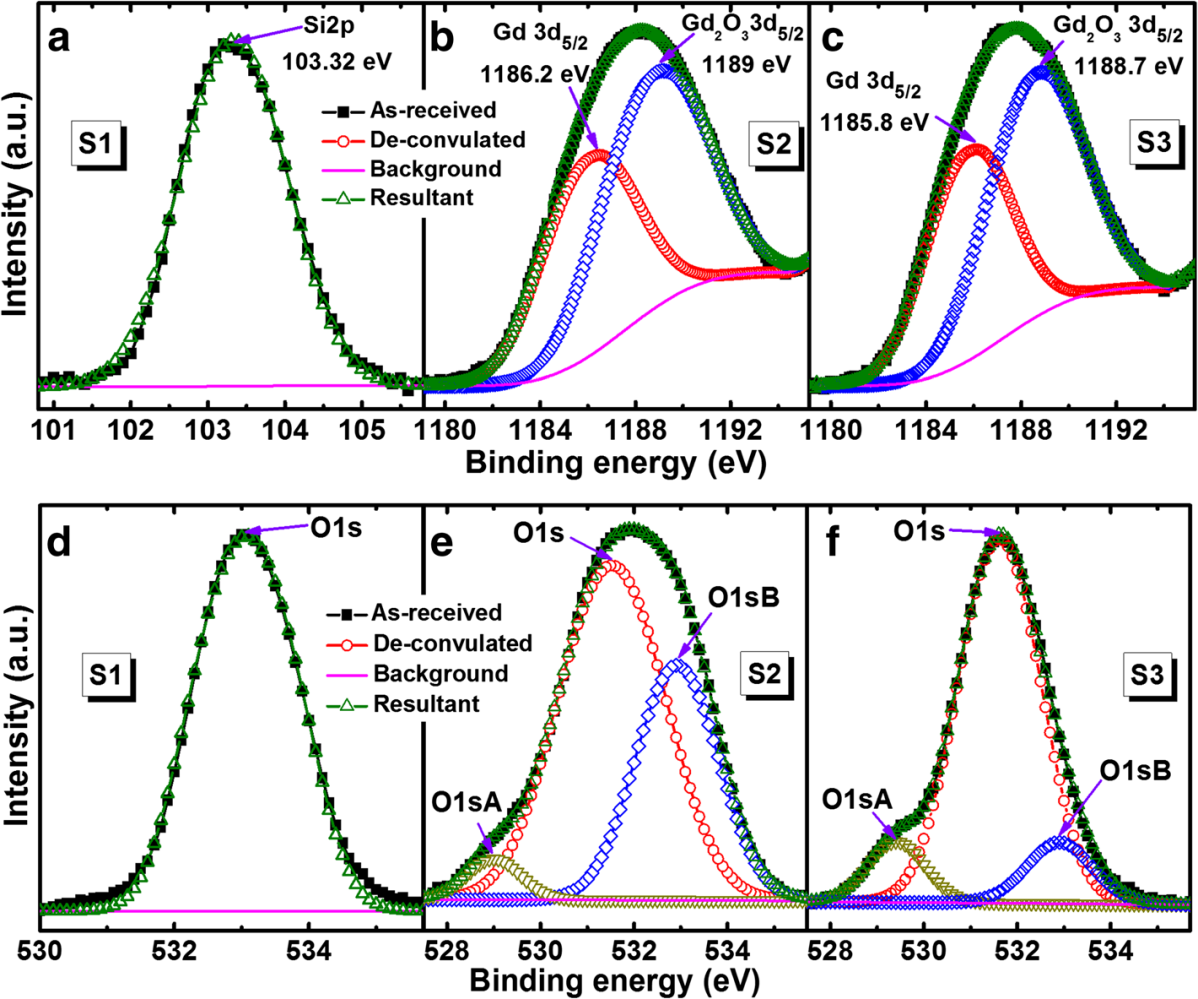
XPS characteristics of Si*2p* for **a **S1, **b** S2, and **c** S3 samples. Corresponding O*1s* spectra of **d** S1, **e** S2, and **f** S3 samples are shown. The S3 film shows higher Gd/Gd_2_O_3_ ratio or oxygen deficient and higher OH group which helps to sense H_2_O_2_

Figure 2 shows the cross-sectional TEM images of the S2 and S3 sensors. The thickness of SiO_2_ is 41.2 nm (Fig. 2a), and the thickness of the GdO_*x*_ film is 3.3 nm (Fig. 2b). The TEM image of the S3 sensor shows that the thickness of SiO_2_ is 41.5 nm (Fig. 2c) and the thickness of the GdO_*x*_ film is 14.8 nm (Fig. 2d). Therefore, the thickness of SiO_2_ is 40 ± 2 nm and the thickness of GdO_*x*_ is 15 ± 0.5 nm. The thicker GdO_*x*_ film shows clearly polycrystalline grains and its boundary [29, 30], which will help to detect H_2_O_2_. Elemental composition of the SiO_2_ and GdO_*x*_ films is observed by XPS, which is shown in Fig. 3. The peak binding energy of Si*2p* spectra for the S1 sample is 103.35 eV (Fig. 3a), which is similar to the reported value of SiO_2_ at 103.58 eV [31]. The spectra are fitted by Shirley background subtraction and Gaussian/Lorentzian functions. The Si*2p* spectrum shows one characteristic peak after de-convolution. Similarly, one characteristic peak of O*1s* centered at 531.5 eV is also observed (Fig. 3d). Lower values of full-width half-maximum (FWHM) are found to be 1.84 and 1.64 eV for the Si*2p* and O*1s* spectra, respectively. The ratio of O:Si is 1.84, which signifies the stoichiometric SiO_2_. An XPS spectrum of GdO_*x*_ shows Gd*3d*_3/2_ and Gd*3d*_5/2_ doublet with binding energy of 1220.5 and 1188.3 eV, respectively (not shown here). However, peak binding energies of Gd*3d*_3/2_ and Gd*3d*_5/2_ spin-orbits are reported as 1218 and 1186 eV, respectively [32]. XPS spectra of Gd*3d*_*5/2*_ core-level electrons are 1189 eV for S2 (Fig. 3b) and 1188.7 eV for S3 (Fig. 3c) samples, which are identified to be Gd_2_O_3_*3d*_5/2_ or Gd_2_O_3_ films. Corresponding lower binding energy peaks at 1186.2 and 1185.8 eV indicate the metallic Gd*3d*_5/2_ peaks for the S2 and S3 samples, respectively. The area ratios of Gd/Gd_2_O_3_ are found to be 0.64:1 and 0.69:1 for the S2 and S3 samples, respectively, which show higher percentage of Gd in the S3 samples owing to polycrystalline grains. However, the O*1s* core-level spectra show three distinct peaks for the S2 (Fig. 3e) and S3 (Fig. 3f) samples. The strong peaks at 531.5 eV correspond to the oxygen in the Gd_2_O_3_ film, whereas lower (O*1s* A) and higher (O*1s* B) binding energy peaks centered at 529 and 532.9 eV are attributed to the hydroxyl (OH^−^) and carbonate groups in Gd_2_O_3_ films, respectively [33, 34]. Moreover, the lower binding energy peak corresponds to Gd-O bonding or GdO_*x*_ [35]. The area ratios of O*1s* A and O*1s* B with respect to O*1s* are 0.04:1 and 0.48:1 for the S2 samples whereas those values are 0.08:1 and 0.1:1 for the S2 samples, respectively. Therefore, the S2 samples show higher percentage of O*1s* B owing to higher carbonate groups in the GdO_*x*_ films, which is insensitive to H_2_O_2_ sensing. On the other hand, the S3 samples have higher percentage of O*1s*A owing to higher OH^−^ and higher Gd content in Gd_2_O_3_ film, i.e., GdO_*x*_ film. So, oxygen can be bonded loosely with Gd on a polycrystalline grain boundary as well as a thicker GdO_*x*_ film will help to sense H_2_O_2_, which will be explained below.

**Fig. 4:**
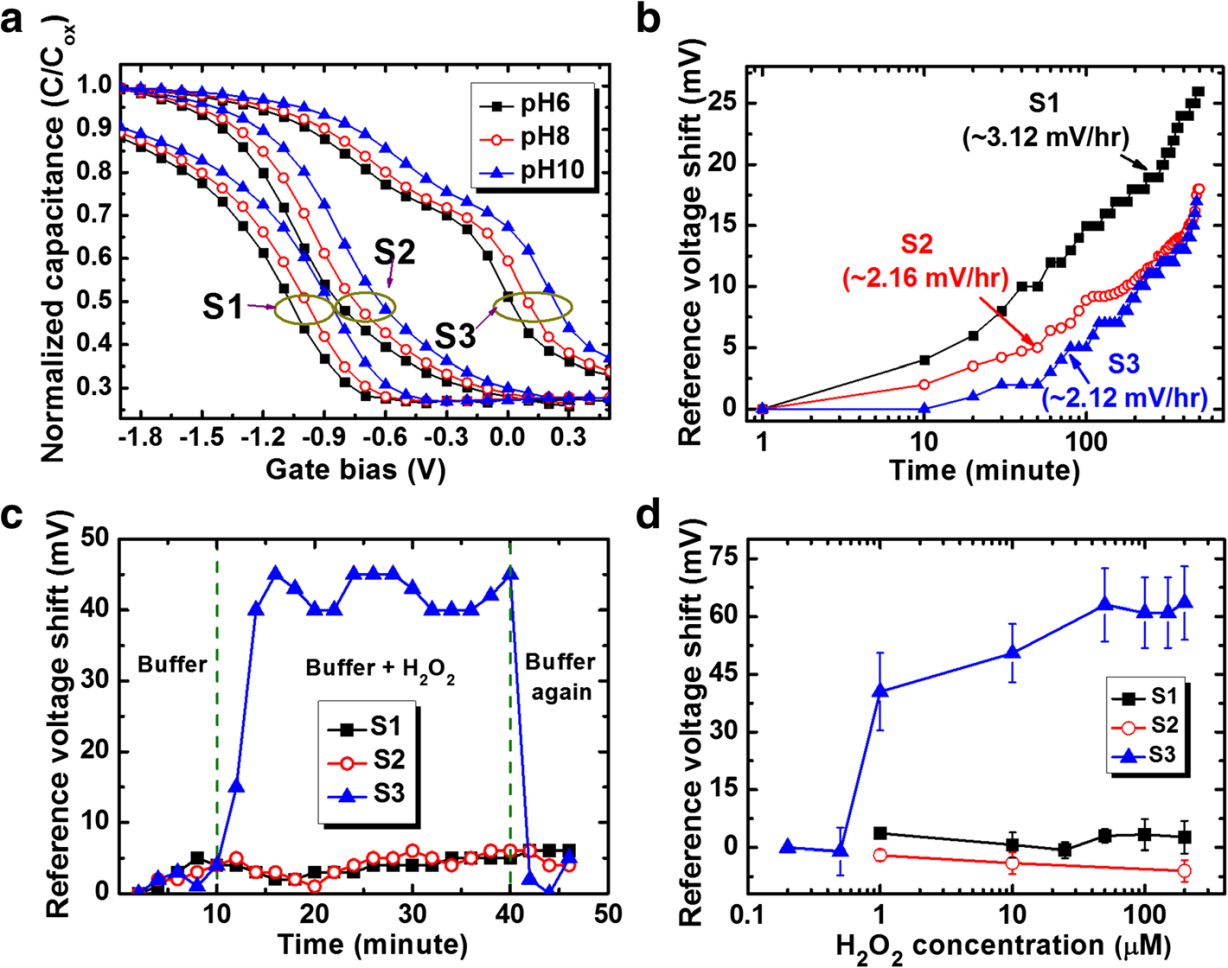
**a** C-V characteristics of the S1, S2, and S3 sensors are shown. **b** Drift rate characteristics of the S1, S2, and S3 sensors. **c** Time-dependent response of H_2_O_2_ and **d** reference voltage shift vs. H_2_O_2_ concentration for all sensors

Figure 4a shows the C-V characteristics with pH values from 6 to 10 for the S2 and S3 sensors. The V_r_ values of the S2 sensors are −0.84, −0.75, and −0.63 V for pH 6, 8, and 10, while those values are 0.01, 0.1, and 0.23 V for the S3 sensors, respectively. The V_r_ values of the S3 sensor are shifted towards the positive direction and are lower than the V_r_ values of the S2 sensors. This is due to lower oxide charges for the thicker GdO_*x*_ membrane (55 vs. 43 nm [36]) and polycrystalline grains with higher OH^−^ ions (Fig. 3f). The pH sensitivity values are found to be 51.2 and 54.2 mV/pH for the S2 and S3 sensors, respectively, which are higher than the pH sensitivity of approximately 35 mV/pH from pH 2 to 10 [28, 37] and 42 mV/pH from pH 6 to 10 for the S1 sensors. The pH sensitivity of a 30-nm-thick GdO_*x*_ membrane is approximately 51.7 mV/pH (not shown C-V curves), which is slightly lower than the S3 sensors. The pH sensitivity value of our GdO_*x*_ membrane is comparable with other reported values of 48.29 mV/pH by Wang et al. [27], 64.78 mV/pH by Chang et al. [38], and 55 mV/pH by Yang et al. [39]. However, the S3 sensors show the lowest drift rate as compared to the S1 and S2 sensors (2.12 mV/h vs. 3.12 mV/h and 2.16 mV/h), as shown in Fig. 4b. The drift characteristics were measured a long time up to 500 min at pH 7 buffer solution. Considering a low drift rate (2.12 mV/h), the pH detection limit of the S3 sensors is 0.039 pH, which is due to high pH sensitivity. It is interesting to note that the GdO_*x*_ membrane will detect H_2_O_2_. Figure 4c shows the time-dependent response of H_2_O_2_ for the S3 sensors. A negligible V_r_ shift is observed for pH 7 buffer solution up to 10 min. By including H_2_O_2_ with a concentration of 1 μM, a good V_r_ shift of approximately 40 mV is observed because of Gd^1+^, Gd^2+^, and Gd^3+^ oxidation states (https://en.wikipedia.org/wiki/Work_function) [40]. On the other hand, both S1 and S2 sensors do not show H_2_O_2_ sensing. When in contact with H_2_O_2_, the Gd^2+^ changes to the Gd^3+^ oxidation state and provides electrons for the reduction of H_2_O_2_. H_2_O as a byproduct is observed (Fig. 1). However, the pH value is unchanged by adding H_2_O_2_ in the buffer solution. A short response time of <2 min is needed without enzyme. After washing out, the sensor does not show any V_r_ shift owing to the reduction from the Gd^3+^ to Gd^2+^ states. Therefore, this sensor can be used repeatedly for H_2_O_2_ sensing. Based on our knowledge, this is the first ever report of H_2_O_2_ detection with a polycrystalline GdO_*x*_ membrane. Basically, the oxidation/reduction of the GdO_*x*_ material in contact with H_2_O_2_ with buffer solutions is responsible for the V_r_ shifting, which is shown by chemical reactions below.

1 $$ \mathrm{G}\mathrm{d}\leftrightarrow {\mathrm{Gd}}^{2+}+2{e}^{-}\leftrightarrow {\mathrm{Gd}}^{3+}+3{e}^{-} $$

2 $$ {\mathrm{H}}_2{\mathrm{O}}_2+{e}^{-}\leftrightarrow {\mathrm{O}\mathrm{H}}^{-}+{\mathrm{O}\mathrm{H}}^{\ast } $$

3 $$ {\mathrm{OH}}^{\ast }+{e}^{-}\leftrightarrow {\mathrm{OH}}^{-} $$

4 $$ 2{\mathrm{OH}}^{-}+2{\mathrm{H}}^{+}\leftrightarrow 2{\mathrm{H}}_2\mathrm{O} $$

**Fig. 5:**
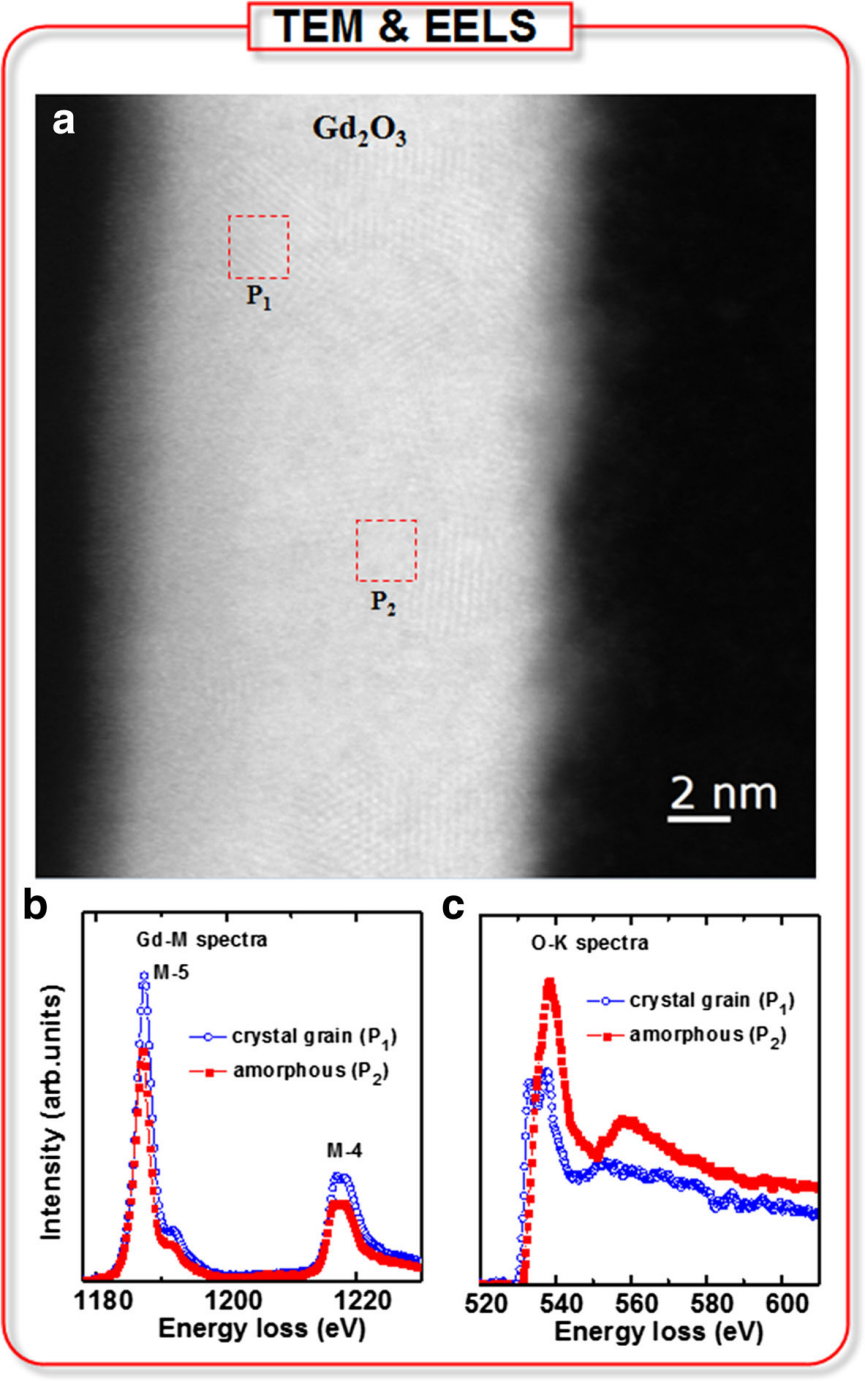
**a** TEM image for EELS spectra of the S3 membranes. The edges of **b** Gd and **c** O-K are plotted for the *P*_1_ and *P*_2_ regions marked on a. The polycrystalline grain corresponds to Gd^2+^ and the grain boundary corresponds to Gd^3+^ oxidation states

By following the above Eqs. (1), (2), (3), and (4), the oxidation state of Gd changes from Gd^2+^ to Gd^3+^. The H^+^ ions are supplied by buffer solutions. The V_r_ shift increases with increasing H_2_O_2_ concentration from 1 to 200 μM because the generation of Gd^3+^ ions increases (Fig. 4d). A moderate sensitivity of 0.13 mV/μM is obtained from a linear range of 1 to 200 μM whereas it is 82 mV/μM from a linear range of 0.5 to 1 μM. Our detection limit of 1 μM is inferior than the published results [9–12, 15, 16, 41–43], comparable with the published results [44–47], and superior than the published results [13, 17, 18, 20, 48–52] in literature by using different sensing methods, as shown in Table 1. Further study is needed to improve the detection limit in the future. However, our sensing method’s surface potential is changed when in contact with H_2_O_2_ because of the catalytic activity of Gd. It is known that Gd_2_O_3_ material is n-type and the energy difference in between the Fermi level and the conduction band (E_c_) is 2.71 eV [53]. The electron affinity of Gd_2_O_3_ is 1.45 eV by considering the conduction band offset of 2.6 eV with Si [54]. The work function of Gd increases from 2.9 eV (https://en.wikipedia.org/wiki/Work_function) to 4.16–4.76 eV [53–55] after oxidation. This suggests that the work function of GdO_*x*_ is modulated by oxidation/reduction or Gd^3+^ concentration as well as the energy band bending of Si is changed. In consequence, the V_r_ is needed to bring Si energy bands to be flat. On the other hand, the S1 and S2 sensors do not show H_2_O_2_ detection because they do not have redox properties. The thinner GdO_*x*_ film (S2) has a smaller crystalline grain with less Gd content (Fig. 3), while the S3 sensor has larger crystalline grain (Fig. 5a) with higher Gd content. Figure 5b shows electron energy loss spectroscopy of Gd measured at polycrystalline grain (P_1_) and amorphous region or grain boundary (P_1_). The regions of P_1_ and P_2_ are marked on Fig. 5a. The edges of the Gd M-4 and M-5 peaks at the P_1_ region are located at 1216.8 and 1187.5 eV, while those values at the P_2_ region are 1216.5 and 1187 eV, respectively. Du et al. [56] have reported the M-4 and M-5 peak values of 1217 and 1185 eV for the Gd(OH)_3_ nanorods. The edges of the O-K peak at both P_1_ and P_2_ regions are located at 538.5 eV, as shown in Fig. 5c, which is close to the reported value of 536.5 eV [56]. It is interesting to note that another peak of crystalline grain (P_1_) is located at 532.9 eV, which is shifted downwards to 3.9 eV. Egerton has reported the reduced energy gap of SiO_*x*_ at the SiO_2_/Si interface with energy shift downwards to 3 eV [57]. In our case, this reduced energy gap is observed in the polycrystalline grain region. Therefore, the crystalline grain is GdO_*x*_ (or Gd^2+^) and the amorphous region or grain boundary is Gd_2_O_3_ (or Gd^3+^). When in contact with H_2_O_2_, the oxidation state of the S3 sensor changes from Gd^2+^ to Gd^3+^ and the crystalline grain takes a major role, which is confirmed by EELS spectra. So, the thicker crystalline GdO_*x*_ membrane can sense H_2_O_2_ repeatedly which will be useful to detect human disease in the near future.

**Table 1:** Comparison of linear range and detection limit of H_2_O_2_ published in literature [9–13, 15–18, 20, 41–52]

Sensing materials	pH value	Linear range (μM)	Detection limit (μM)
MoS_2_ NP [15]	7.4	5–100	0.002
WS_2_ NS [10]	7.4	–	0.002
Pt-Pd-Fe_3_O_4_ [16]	7.4	0.02–0.1, 2–14,000	0.005
Pt-Pd/rGO [11]	7.0	0.1–37.6	0.01
Au NP [12]	7.0	2–5000	0.01
Pt NP [9]	7.2	3–300	0.03
rGO [41]	7.0	0.05–1500	0.05
Au/C/Pt [42]	7.0	9.0–1860, 1860–7110	0.13
Au NP [43]	6.8	3–605	0.18
Ag NP [44]	7.5	100–10,000	0.88
GS/CeO_2_-ZnO NP [45]	7.0	2–20,000	1.1
Pt-Pd and Pt-Ir [46]	7.4	2.5–125	1.2
Pt NP [47]	6.9	5–2000	1.23
CeO_2_ NP/N-rGO [48]	7.0	1.8–920.8	1.3
CuO [13]	7.0	10–13,180	1.6
Ag NPs/PPy/Fe_3_O_4_ [49]	7.2	5–11,500	1.7
Pd NP [17]	7.4	2–1300	2
Co_3_O_4_ NW [18]	7.4	15–675	2.4
Carbon dots [20]	7.4	3–300	3
Se/Pt [50]	7.0	10–15,000	3.1
Ag NP [51]	7.0	25–500, 500–5500	10
Co-Mn [52]	7.2	100–25,000	15
GdO_*x*_ in EIS structure (this work)	7.0	1–200	1

*NP* nanoparticle, *NS* nanosheet, *rGO* reduced graphene oxide, *GS* graphene sheet, *NW* nanowire

## Conclusions

Higher pH sensitivity (54.2 m/pH) and the enzyme-free H_2_O_2_ sensing characteristics have been investigated by using 15-nm-thick GdO_*x*_ membranes for the first time. The polycrystalline grain and thickness of the GdO_*x*_/SiO_2_ film have been observed by TEM image. XPS characteristics of the S3 membrane show higher Gd/Gd_2_O_3_ ratio than the S2 membrane (0.69/1 vs. 0.64/1). The S3 membrane shows GdO_*x*_ and higher OH content in the crystalline grain, which help to sense H_2_O_2_ whereas both S1 and S2 sensors do not show H_2_O_2_ detection. Therefore, a larger polycrystalline GdO_*x*_ grain has oxidation/reduction properties when in contact with H_2_O_2_, which is confirmed by EELS. During oxidation, the Gd^2+^ changes to the Gd^3+^ state and the amount of Gd^3+^ ions increases with increasing H_2_O_2_ concentration from 1 to 200 μM. A low defection limit of 1 μM is obtained owing to the catalytic effect of Gd. The time-dependent response and the sensing mechanism of H_2_O_2_ have been explored. Due to the short time detection of H_2_O_2_ in the EIS structure, this novel GdO_*x*_ sensing membrane paves a way to diagnose other diseases of the human body in the near future.
